# Impact of chronic obstructive pulmonary disease on passive viscoelastic components of the musculoarticular system

**DOI:** 10.1038/s41598-021-97621-9

**Published:** 2021-09-10

**Authors:** Maria Stella Valle, Antonino Casabona, Eugenia Di Fazio, Claudia Crimi, Cristina Russo, Lucia Malaguarnera, Nunzio Crimi, Matteo Cioni

**Affiliations:** 1 Laboratory of Neuro-Biomechanics, Department of Biomedical and Biotechnological Sciences, School of Medicine, University of Catania, Catania, Italy; 2 Respiratory Medicine Unit, “Policlinico Vittorio Emanuele-San Marco” University Hospital, Catania, Italy; 3 Section of Pathology, Department of Biomedical and Biotechnological Sciences, University of Catania, Catania, Italy; 4 Department of Clinical and Experimental Medicine, University of Catania, Catania, Italy; 5 Gait and Posture Analysis Laboratory, “Policlinico Vittorio Emanuele-San Marco” University Hospital, Catania, Italy

**Keywords:** Diseases, Medical research

## Abstract

Chronic obstructive pulmonary disease (COPD) produces skeletal muscle atrophy and weakness, leading to impairments of exercise performance. The mechanical work needed for movement execution is also provided by the passive tension developed by musculoarticular connective tissue. To verify whether COPD affects this component, the passive viscoelastic properties of the knee joint were evaluated in 11 patients with COPD and in 11 healthy individuals. The levels of stiffness and viscosity were assessed by means of the pendulum test, consisting in a series of passive leg oscillations. In addition, to explore the contribution of passive tension in the mechanical output of a simple motor task, voluntary leg flexion–extension movements were performed. Patients with COPD showed a statistically significant reduction in stiffness and viscosity compared to controls. Voluntary execution of flexion–extension movements revealed that the electromyographic activity of the Rectus Femoris and Biceps Femoris was lower in patients than in controls, and the low viscoelastic tension in the patients conditioned the performance of active movements. These results provide novel insights on the mechanism responsible for the movement impairments associated with COPD.

## Introduction

Chronic Obstructive Pulmonary Disease (COPD) is the name for a group of lung conditions that causes alterations in ventilatory mechanics, airway limitation and alveolar abnormalities. These conditions result in symptoms such as fatigue and shortness of breath. Symptoms and rate of clinical progression of the disease may vary widely among individuals, but all patients with stable COPD also exhibit reduced peripheral muscle endurance and great fatigability^1–3^. This uncomfortable condition leads to limitation of physical activity, with the consequence of further alterations in muscle function and a progressive decline in their ability to perform exercise.

Biochemical and histological studies on the effects of COPD on peripheral skeletal muscle have shown enzymatic changes and mitochondrial abnormalities^1^, a reduction in the cross-sectional area^4,5^ and changes in the composition of the type of fibers switching from slow‐oxidative to fast‐glycolytic fiber type^6,7^. These dysfunctions exacerbate fatigability resulting in muscle weakness and atrophy, especially in lower limb muscles^8,9^. Limited oxygen supply and reduced lower extremity muscle strength create a point of vulnerability that results in a poor functional capacity of locomotor muscles and in a reduced ability in performing normal daily activities, such as walking or upright standing^10–13^.

While impaired muscle function is certainly a crucial factor in producing motor task disabilities in patients with COPD, changes in passive viscoelastic elements in muscle and joint structures could also play a role in determining the movement dysfunctions associated with COPD. Passive tensions can be produced by the connective tissue integrated into the muscle structure (perimysium, epimysium and endomysium), tendons, non-contractile sarcomere proteins (titin and nebulin) and tissues associated with the joints, such as ligaments or capsules. These structures develop a passive viscoelastic tension that can counterbalance or assist muscle active tension and contribute to the level of joint stability and mobility^14–16^.

Although the potential influence of the viscoelastic musculoarticular components on movement performance is well-established^14–18^, to our knowledge no studies have been conducted to explore possible changes in muscle and joint passive tension in the lower limbs of patients with COPD. Thus, the purpose of this study was to provide a basic quantification of the level of passive elastic stiffness and viscosity in patients with COPD.

We addressed this goal by adopting two types of measurement protocols based on repetitive knee flexion–extension movements over the sagittal plane. In a first set of measurements, passive viscoelastic tension was evaluated by using the Wartenberg pendulum test, consisting in passive leg oscillations performed under the simply drive of gravitational torque^19^. A second protocol was designed to evaluate the mechanical performance during a set of leg flexion–extensions executed by voluntary muscle contractions. The combination of passive and active spatiotemporal measurements of leg oscillations allowed us to assess the contribution of passive components when these interact with the muscular contraction in performing a simple voluntary motor task.

Overall, we hypothesize that information on changes in the level of passive viscoelastic tension in patients with COPD may provide novel insights into the mechanisms responsible for the alteration in motor abilities caused by this disease.

## Material and methods

### Ethical statement

This study was approved by the local ethics committee of Catania University Hospital “Policlinico Vittorio Emanuele-San Marco” (n° 134/2019/PO), and all participants provided written consent to participate, before starting the study. All procedures were performed according to the Declaration of Helsinki.

### Participants

The criteria of inclusion to participate in the study were the following: diagnosis of COPD according to Global Initiative for Chronic Obstructive Lung Disease (GOLD) criteria^20^, no disease exacerbation within the 4 weeks prior to the beginning of the study. The following exclusion criteria were adopted: presence of heart diseases, musculoskeletal traumas, diabetes, rheumatologic diseases, inability to relax muscles during passive movements.

We enrolled 14 non-hospitalized patients with moderate to severe COPD, related to the Respiratory Medicine Unit, “Policlinico Vittorio Emanuele-San Marco” University Hospital, (Catania, Italy). None of them had had pulmonary rehabilitation sessions, before carrying out the tests. The study also included a control group of 13 healthy individuals from the medical and nursing staff at the same hospital. Until the time of recording, none of them had had problems of their cardio-respiratory system. They are non-smoking, sedentary and with normal lung function and comparable age, gender and anthropometric measures to the patient group. Three patients refused to participate as they did not perceive the study as relevant, whereas two healthy participants were excluded from the study because of their inability to relax their muscles during passive movements. The anthropometric data from the two groups, summarized in Table 1, showed no statistically significant differences: age (ys): COPD, 63.8 ± 11.9, Control 64.4 ± 5.8, p = 0.875; weight (kg): COPD, 69.4 ± 13.7, Control 71.3 ± 12.6, p = 0.736; height (cm): COPD, 164.9 ± 9.7, Control 164.7 ± 8.4, p = 0.963.

**Table 1:** Anthropometric data for the patients with COPD and healthy individuals enrolled in this study.

#	Patients				Healthy			
	Gender	Age (ys)	Weight (kg)	Height (cm)	Gender	Age (ys)	Weight (kg)	Height (cm)
1	F	73	64	145	M	65	63	159
2	M	50	87	183	F	68	83	172
3	M	74	60	170	M	60	85	179
4	F	39	52	156	M	67	90	174
5	F	80	67	165	M	63	65	163
6	M	68	51	171	M	78	68	169
7	F	72	75	166	F	68	70	163
8	F	64	79	167	M	61	65	165
9	M	56	80	168	F	63	62	163
10	F	60	58	157	F	58	84	153
11	M	66	90	166	F	58	49	152

All patients underwent spirometry and the following parameters of lung volumes were considered to make the diagnosis: forced vital capacity (FVC), forced expiratory volume in one second (FEV1) and FEV1/FVC ratio. To assess the severity of functional impairment, the Diffusing capacity for carbon monoxide (D_LCO_)^21^ and the modified Medical Research Council (mMRC) Dyspnea scale^22^, were used. Table 2 shows a summary of the clinical evaluations performed on the patients.

**Table 2:** Clinical data for the patients with COPD enrolled in this study.

Patients	Smoker	Years of smoking	GOLD group	FEV1 (%)	FVC (%)	FEV1/FVC (%)	D_LCO_ (%)	mMRC
#1	NO	–	C	113	114	67	97	1
#2	EX	50	A	64	100	41	105	0
#3	YES	50	A	75	62	63	122	1
#4	NO	–	D	39	56	54	97	2
#5	EX	40	C	75	113	47	94	1
#6	EX	40	B	27	86	21	35	2
#7	EX	10	D	81	89	65	82	1
#8	EX	40	B	70	91	56	86	2
#9	EX	30	D	33	47	52	55	4
#10	NO	–	D	77	85	67	73	1
#11	EX	20	C	69	99	47	89	2

GOLD: Global Initiative for Chronic Obstructive Lung Disease; FEV1: forced expiratory volume in one second; FVC: forced vital capacity; FEV1/FVC: ratio between two terms; D_LCO:_ diffusing capacity for carbon monoxide; mMRC: modified Medical Research Council Dyspnea scale.

### Procedures and data processing

All the patients and the healthy individuals performed the passive and the active protocols sitting in a comfortable position with the trunk inclined approximated 40° from the horizontal plane.

#### Passive movement protocol: pendulum test

**Figure 1:**
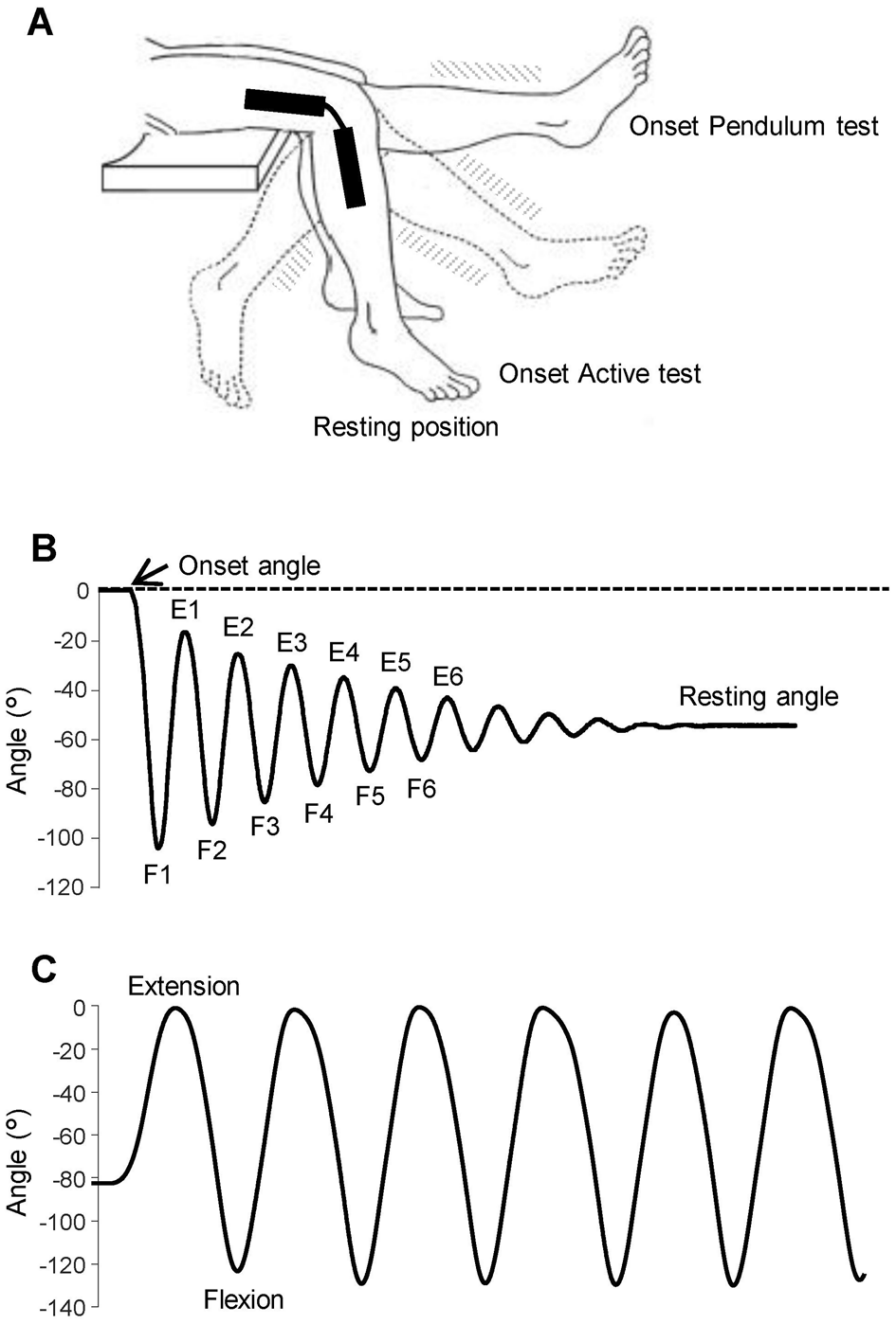
Experimental setup and typical kinematic trajectories in passive and active protocols. (**A**) Leg oscillations during passive pendulum test and active flexion–extension movements. (**B**) Knee angular displacement during the pendulum test, showing onset angle corresponding to the maximum leg extension, resting angle corresponding to the leg resting position and the first 12 hemicycles (flexions: F1, F2, .. F6; extensions E1, E2, .. E6). (**C**) Knee angular displacement during voluntary flexion and extension movements with the onset angle corresponding to the resting position. An electrogoniometer recorded the angular displacements of the knee and two couples of surface electrodes recorded the EMG activity of the Rectus Femoris and Biceps Femoris.

Participants performing the pendulum test (Fig. 1A,B) were instructed to relax, to prevent significant changes in muscle tone or the occurrence of systematic phasic muscle activity. To ensure that the measurements captured during leg movements were a reliable estimation of changes in the passive viscoelastic component of the knee joint, a continuous electromyographic (EMG) recording from the Rectus Femoris (RF) and the Biceps Femoris caput longus (BF) was performed. Trials showing the level of EMG activity consistently above the baseline level present before the leg oscillations were excluded and repeated. Likewise, the trials with recurrently occurring phasic muscle activity and in synchrony with the movements were excluded.

After a clear explanation and demonstration of the procedure, the experimenter lifted the dominant lower limb of each participant to the horizontal starting position and, after checking the relaxation of the limb, left the leg swing passively between flexions and extensions until it stopped at the resting position (Fig. 1A). This procedure was repeated 10 times.

Angular displacements of the swing movements were recorded by an electrogoniometer placed on the lateral surface of the knee joint as illustrated in Fig. 1A. Subsequently, kinematic data were filtered using a low-pass, zero-lag second-order Butterworth filter with 5 Hz cutoff frequency. The EMG data were gathered by surface electrodes held in place by medical tape placed over the RF and the BF according to the SENIAM protocol^23^. The correct position of the electrodes was checked by visual inspection of the EMG signal. The kinematics and EMG measurements were recorded and synchronized by using a portable device (POCKET-EMG by Bioengineering Technology and System–BTS, Garbagnate Milanese, Italy). All the signals were sampled at 1000 Hz, with kinematic data resampled at 200 Hz for further processing. The EMG signal was amplified and high-pass filtered (20 Hz) to remove low frequency artifacts produced by electrode movements.

The spatiotemporal analysis and EMG processing were based on the following acquired parameters (Fig. 1B):

- onset angle, corresponding to the maximum extension of the leg, before the onset of the first flexion;
- resting angle, corresponding to the angle at rest, at the end of leg oscillations;
- peak angle amplitude, peak velocity and time from onset at the reversal points for each flexion (F1–F3) and extension (E1–E3) hemicycle, for the first three cycles of oscillations;
- EMG area during each hemicycle obtained by computing the integral of EMG signal after a full wave rectification and the application of a low-pass filter (10 Hz, zero-lag, 4th-order Butterworth filter).

Lastly, we used kinematic and anthropometric data to estimate the viscosity (B) and stiffness (K) coefficients for the first three flexions and extensions of the knee joint. The following equation was used as a model for the pendulum motion:

1 $$J{ \ddot{\theta }} + {\text{B}}{\dot{\theta }} + {\text{K}}\theta + mgl_{c} {\text{sin}}\theta = 0$$

where θ represents the angle of oscillation of the leg; *J* is the moment of inertia of the leg-foot complex with respect to the axis of rotation around the knee; B is the viscosity coefficient; K the stiffness coefficient; *mgl*_*c*_ represents the gravitational torque produced by gravity on the knee, where *m* is the mass of the leg-foot complex, *l*_*c*_ is the distance between the center of mass of the leg-foot complex and the axis of rotation around the knee and *g* is gravity acceleration. The value of *m* was estimated as a percentage of the total body weight on the basis of the anthropometric data reported by Winter^24^. The value of *l*_*c*_ was measured from the head of fibula to the ground with the participant in a standing position. Starting from this model, K and B were computed following the procedure described in detail in Casabona et al*.*^25^.

The values of kinematic data, stiffness and viscosity were normalized by the gravitational torque.

#### Active movement protocol

Of all the participants who completed the pendulum test, one patient did not carry out the second test due to unavoidable personal commitments. Hence, after an appropriate rest break, ten out of the eleven patients and eleven healthy participants performed the active oscillation test (Fig. 1A,C).

After the pendulum test was completed, each participant was required to remain in the resting position and execute six voluntary and consecutive leg oscillations starting from an extension movement (Fig. 1A,C). Participants were asked to accomplish the movements applying the muscle strength necessary to bring the leg to maximum extension and flexion for each cycle.

The quantification of the kinematics and the EMG activity related to the active oscillating movements was made computing the average velocity and the average EMG area over the six flexions and the six extensions, separately. Kinematics and EMG signals were acquired, sampled and filtered as in the pendulum test. The average level of EMG background recorded 500 ms before the onset of the first flexion was subtracted from the EMG activity acquired during the leg oscillations. No amplitude normalization was performed for the EMG amplitude, such as % of EMG activation during maximum voluntary contraction, to avoid cancelling out the EMG amplitude differences between the two groups. Moreover, considering the dynamics of our active task, two issues would arise: first, maximum voluntary contraction would correspond to the activation of all muscles used in the task, while we recorded the EMG from only two single muscles; second, the length of muscle changes continuously during flexion–extension movements, while maximum voluntary contraction would be measured at a fixed muscle length.

All data and signal processing was performed using Matlab version R2020a (Mathworks Inc., Natick, MA, USA).

### Statistical analysis

Preliminary power analysis was performed to establish an appropriate sample size. Power analyses were performed by using G*Power version 3.1.9.7^26^ and input parameters for the sample size computation were based on the data from our previous papers^25,27^.

Partial eta squared (η^2^_p_) was used to estimate the sample size for the analysis of variance (ANOVA) with repeated measures and within-between interactions (data from^27^). A total sample size of eight participants for each group was obtained performing an a priori power analysis computation with the following input parameters: η^2^_p_ = 0.3; 2 tails; α = 0.05; power = 0.95; number of groups, 2; number of repetitions, 6; correlation among repeated measures, 0.5; non-sphericity correction, 1.

For t-test comparisons, the sample size estimation was based on the differences between two independent means considering the values of means and variances of knee angular amplitudes observed during previously performed pendulum test^25^. A sample size of seven participants for each group was obtained with a priori power analysis and performing a computation by using the following input parameters; 2 tails; α = 0.05; power = 0.95. Therefore, eleven participants for each group for the passive test and ten patients and eleven healthy individuals for the active test, were considered a sufficient quantity for the results to be meaningful.

The data were preliminarily subjected to the Shapiro–Wilk test to verify the presence of a normal distribution of the sample, and to the Levene’s test to verify the homogeneity of the variances between the groups.

Assuming a normal distribution of data, two-way analysis of variance (ANOVA) for repeated measures was used for the pendulum test data, considering the variations in viscosity and stiffness coefficients during the first six hemicycles as “*within”* repeated factor (hemicycle) and the variations between the two groups as “*between”* factor (group). Furthermore, the interaction between the two factors was evaluated. The statistical evaluations of changes in kinematics and EMG activity during the active oscillation test, were based on a two-way ANOVA with groups (group) and flexion/extension movements (movement direction) as main factors, with movement direction as repeated factor. For both passive and active protocols, the comparison between the two groups for each single parameter was performed using the Student’s t-test. When appropriate, the post hoc comparisons were carried out with the t-test corrected by Bonferroni procedure. The magnitude of the effect was evaluated by partial eta squared (η^2^_p_).

To determine the level of contribution of muscle activation and passive kinematic parameters in predicting the active flexion–extension performance, the following multivariate linear model was used:

2 $$\mathrm{V} = {\beta }_{0} + {\beta }_{1}\mathrm{K} + {\beta }_{2}\mathrm{B} + {\beta }_{3}\mathrm{RF} + {\beta }_{4}\mathrm{BF} + \varepsilon$$

where V is the average velocity over the hemicycles, representing the active movement performance; K and B are stiffness and viscosity estimated by the pendulum test; RF and BF are the area of EMG activity recorded for the Rectus Femoris and Biceps Femoris over the hemicycles during the active protocol; *β*_*0-4*_ are the standardized partial coefficients and *ε* is the residual error.

Starting from this model, we performed a backward stepwise regression to exclude non-significant independent variables from the model and obtained a final model with the variables predicting the V with the best statistical significance. The estimation of the sample size in relation to this regression model was computed considering a linear multiple regression with fixed model and coefficient of determination (*R*^2^) increase. An a priori type of power analysis was performed considering a partial coefficient of determination (*r*^2^) corresponding to 0.5, that is, each independent variable should explain 50% of the total variance in the dependent variable. The sample size computation was based on the following input parameters; α = 0.05; power = 0.8; total number of predictors, 4; number of tested predictors 1–4. The estimated total sample size ranged from 12 to 18 individuals, depending on whether 1 to 4 predictors were included in the final model. Given that the estimated sample size differs to some extent from the number of participants included in this paper, we will pay attention to the data obtained from the multiple regression analysis and provide a complete set of statistical tests to evaluate the strength of correlation and the levels of significance.

The strength of the linear correlation between the model and the dependent variable was estimated by the correlation coefficient (*R*), *R*^2^ and adjusted *R*^2^ (*aR*^2^). When final models resulted in more than one independent variable, multicollinearity among the independent variables was examined by computing the Variance Inflation Factor (VIF).

ANOVA was adopted to test the statistical significance of the regression models. When the final model included two or more variables, the contribution of each variable in predicting the dependent variable was estimated by the partial coefficient of correlation (*r*) and *r*^2^.

For all the statistical tests, the significance level α was established at 0.05. Statistical analysis was performed by using SPSS version 26 (SPSS, Inc., Chicago, IL, USA, IBM, Somers, NY, USA).

## Results

### Passive oscillations of the leg

**Figure 2:**
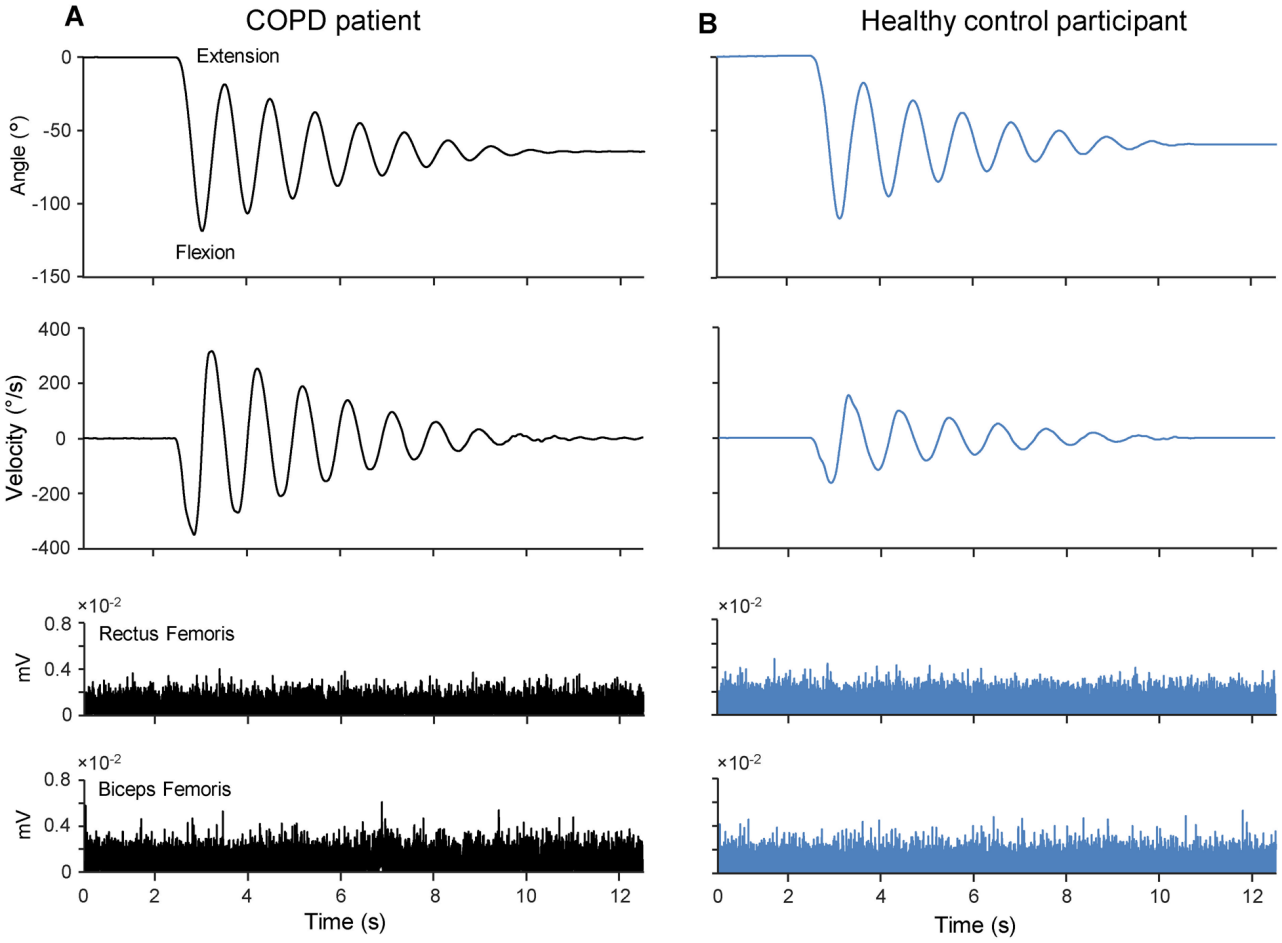
Representative examples of kinematic and electromyographic traces during pendulum test. Angular displacement, velocity, rectified EMG activity of Rectus Femoris and Biceps Femoris muscles, recorded in one patient with COPD (**A**) and one healthy control participant (**B**).

Two examples of kinematic and EMG traces recorded during single trials in one healthy person and one COPD patient are illustrated in Fig. 2. The kinematic data show that the patient with COPD (Fig. 2A) compared with the healthy person (Fig. 2B) exhibited larger peaks of angular displacement and velocity, resulting in a longer duration of oscillations. In both examples, the EMG traces of RF and BF muscles were characterized by a constant low tonic activity, with no phasic burst.

**Figure 3:**
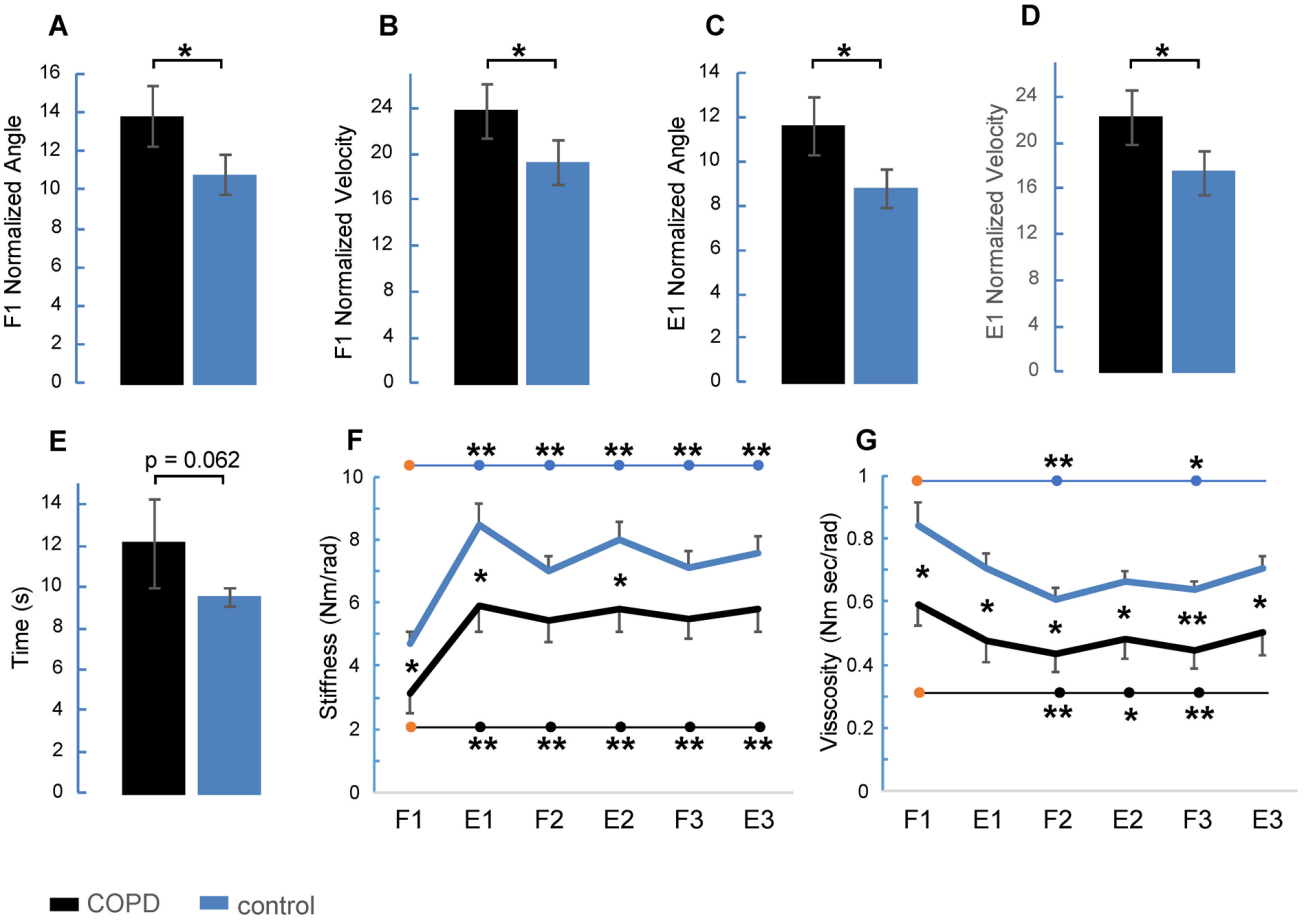
Statistical analysis of kinematic and viscoelastic data. Comparisons between COPD (black) and control (blue) groups for the following parameters: normalized angle (**A**) and velocity (**B**) during the first flexion; normalized angle (**C**) and velocity (**D**) during the first extension; total time of pendular leg oscillations (**E**). Changes in stiffness (**F**) and viscosity (**G**) estimated for COPD (black) and control (blue) groups over the first six hemicycles. Data are expressed as grand average and standard error. Symbols: *p < 0.05; **p < 0.01.

Comparing the two samples of participants, we found that COPD and control groups differed significantly for all kinematic parameters measured during the first knee flexion–extension movement (Fig. 3A–D). In particular, patients showed higher values than the control group for F1 angle amplitude (Fig. 3A, p = 0.026), F1 velocity (Fig. 3B, p = 0.04), E1 angle amplitude (Fig. 3C, p = 0.016) and E1 velocity (Fig. 3D, p = 0.029). Moreover, the total time of oscillations was longer for the COPD group with respect to the control group, with a marginal statistical significance (Fig. 3E, p = 0.062).

Two-way ANOVA was used to evaluate changes in knee stiffness (Fig. 3F) and viscosity (Fig. 3G). During the first six consecutive hemicycles there was a main effect of group for stiffness (Fig. 3F), with the patients showing lower values than the control group (F_1,20_ = 4.525, p = 0.046, η^2^_p_ = 0.18). A significant effect was also seen by the hemicycle factor (F_2.4,47.8_ = 88.127, p < 0.001, η^2^_p_ = 0.81), with an increase in stiffness from F1 to the following hemicycles. Finally, there was a marginal statistical significance for the interaction between hemicycle and group (F_2.4,47.8_ = 2.658, p = 0.071, η^2^_p_ = 0.12). Along the hemicycles, both the groups showed significant differences between F1 and each of the following hemicycles (p < 0.001), but in the control group, the changes in stiffness over the hemicycles were less attenuated than in the patients. Paired comparisons between groups, for each hemicycle, revealed that there were differences only for F1 (p = 0.048), E1 (p = 0.03) and E2 (p = 0.039).

The changes in viscosity (Fig. 3G) produced a main effect of group (F_1,20_ = 8.112, p = 0.010, η^2^_p_ = 0.29), with lower values observed in the COPD group than in the control group. The viscosity variations were highly significant over the hemicycles (F_2.7,53.2_ = 16.431, p < 0.001, η^2^_p_ = 0.45), but no effects were detected for the interaction between hemicycle and group (F_2.7,53.2_ = 0.794, p = 0.489, η^2^_p_ = 0.04). With respect to stiffness, the level of viscosity showed more gradual changes along hemicycles, with significant differences between F1 and the hemicycles F2 (p = 0.005) and F3 (p = 0.029) for the control group, and between F1 and the hemicycles F2 (p = 0.002), E2 (p = 0.048) and F3 (p = 0.004) for the patients. Significant effects between groups were found for each hemicycle, resulting in a parallel reduction of viscosity for the two groups.

There were no statistical differences between groups and over the hemicycles for the changes in EMG activity of RF and BF muscles.

### Active oscillations of the leg

**Figure 4:**
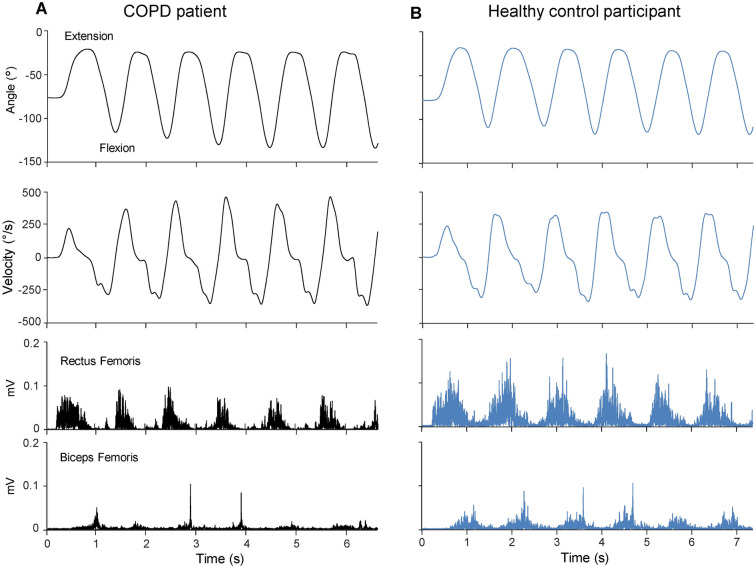
Representative examples of kinematic and electromyographic traces during voluntary flexion–extension movements. Angular displacement, velocity, rectified EMG activity of Rectus Femoris and Biceps Femoris muscles, recorded in one patient with COPD (**A**) and one healthy control participant (**B**).

Knee angular displacement, movement velocity and muscle activation during the active protocol are shown in Fig. 4 for two representative participants. In particular, the values of kinematic parameters were similar comparing the patient with the healthy person, but the level of EMG activations in the patient was lower than in the control participant for both RF and BF muscles.

**Figure 5:**
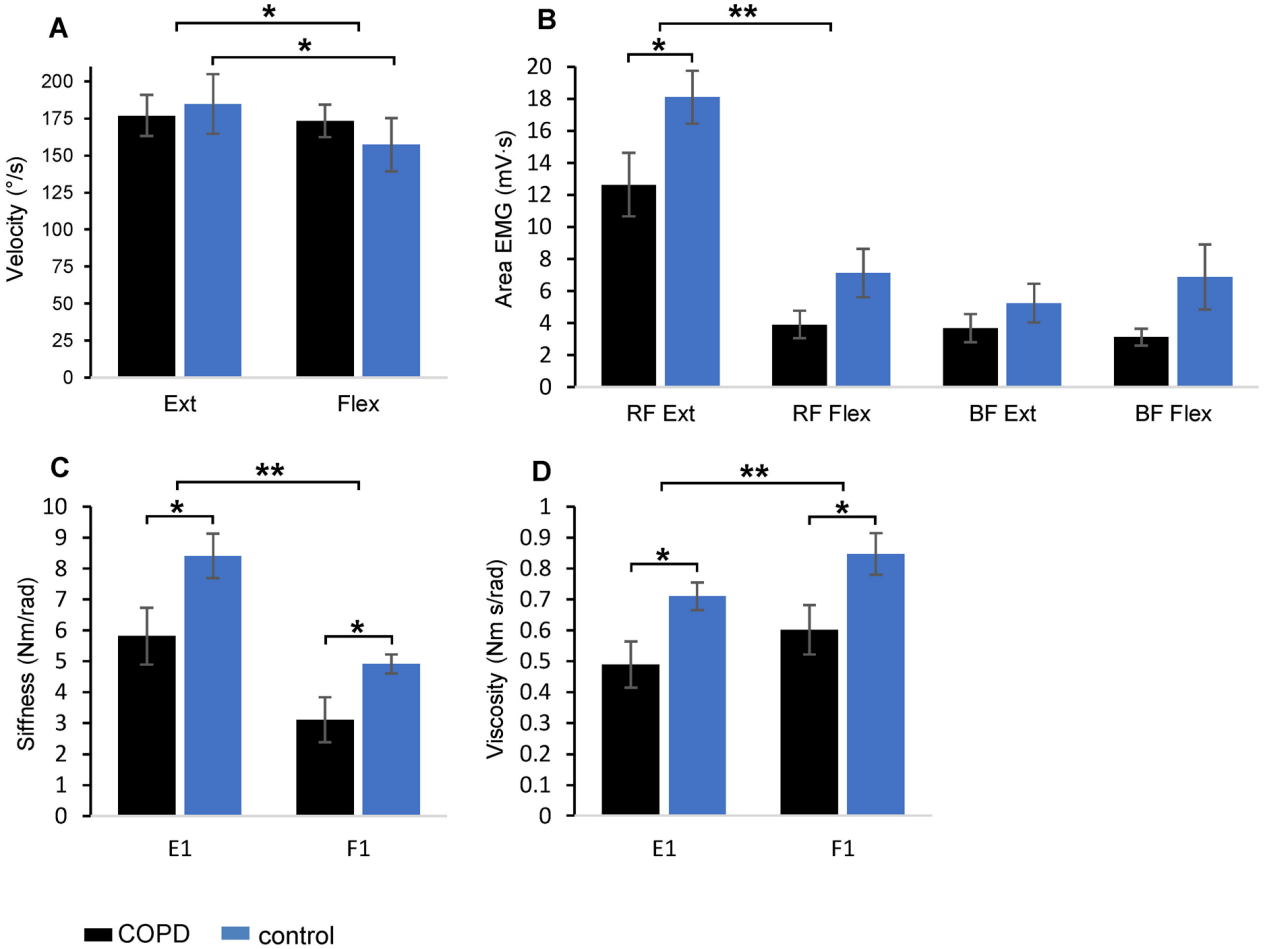
Comparisons between data from the active and passive protocols. The average values of movement velocity (**A**) and EMG area of Rectus Femoris and Biceps Femoris (**B**), computed over the six extension and the six flexion active movements, acquired in COPD (black) and control (blue) groups. Values of stiffness (**C**) and viscosity (**D**) during the first passive flexion and extension of the pendulum test. Abbreviations: *Ext*, active extensions; *Flex*, active flexions, *RF*, Rectus Femoris, *BF*, Biceps Femoris, *F1*, the first passive flexion, *E1*, the first passive extension. Symbols as in Fig. 3.

Analyzing the two samples, extension and flexion movement velocity (Fig. 5A) showed no main effect of group (F_1,19_ = 0.035, p = 0.853, η^2^_p_ = 0.01), but there was a main effect of movement direction (F_1,19_ = 4.919, p = 0.039, η^2^_p_ = 0.21). However, only the control group exhibited statistical differences between the movement directions, with higher velocity in extensions than in flexions (p = 0.017).

Statistically significant changes in EMG activity during the flexion–extension oscillations were focused on the RF muscle during the leg extension movements (Fig. 5B). In this case, there was a main effect of direction (F_1,19_ = 46.699, p < 0.001, η^2^_p_ = 0.71), with both groups exhibiting significant reduction of RF area (patients: p = 0.001; controls: p < 0.001). A main effect of group (F_1,19_ = 6.646, p = 0.018, η^2^_p_ = 0.26) was also detected, but a significant decrease of RF activity was only observed in the group of patients for the extension movements (p = 0.047). The prevalence of the RF EMG activity is not surprising since the RF is a leg extensor muscle and the extension movements were executed against gravity. On the contrary, although the BF muscle acts as a leg flexor, no significant differences were found between movement directions (F_1,19_ = 3.156, p = 0.546, η^2^_p_ = 0.02) or groups (F_1,19_ = 2.572, p = 0.125, η^2^_p_ = 0.13) for this muscle. In this case, the movement was facilitated by the direction of gravity, reducing the involvement of the BF muscle.

The quantification of the contribution of active and passive parameters on determining the observed velocity movements was performed considering the changes in EMG area of RF and BF muscles and the values of stiffness and viscosity measured during the first extension and first flexion of the pendulum test. We used the values of the first two hemicycles for passive parameters (see Fig. 3F,G), since their kinematics were more comparable with the flexion and extension kinematics performed during the six leg oscillations of the active protocol. For an appropriate comparison between passive and active measurements, the data of the first flexion and first extension for stiffness and viscosity were recomputed because one patient did not participate in the active trial. The statistical results of this reworking are summarized in Fig. 5C,D. Stiffness and viscosity showed significant differences between movement direction (stiffness: F_1,19_ = 38.210, p < 0.001, η^2^_p_ = 0.67; viscosity: F_1,19_ = 10.173, p = 0.005, η^2^_p_ = 0.35) and between groups (stiffness: F_1,19_ = 6.896, p = 0.017, η^2^_p_ = 0.27; viscosity: F_1,19_ = 7.246, p = 0.014, η^2^_p_ = 0.28) for both the first extension (stiffness: p = 0.036; viscosity: p = 0.018) and the first flexion (stiffness: p = 0.029; viscosity: p = 0.029).

From the multiple regression model represented by Eq. (2), a backward stepwise processing was adopted to find the best relationship between changes in velocity and changes in passive and active parameters during the extension and flexion voluntary movements. The results of this analysis, reported in Table 3, show that there were suitable levels of correlation strength (see columns related to *R, R*^2^ and *aR*^2^), a good level of statistical significance (see columns related to ANOVA) and, in the final models with more than one independent variable, acceptable range of VIF to conclude that there were no multicollinearity symptoms (see rows related to VIP for models associated with Extension Velocity).

**Table 3:** Backward stepwise regression analysis to quantify the relationships between movement velocity and active and passive parameters.

Dependent variables	Groups	Independent variables					Multiple correlation statistics			ANOVA	
			RF	BF	K	B	*R*	*R*^2^	*aR*^2^	F	p
Extension velocity	COPD	*r*	0.5		− 0.39	− 0.7	0.89	0.79	0.69	7.62	0.018
		*r*^2^	0.25		0.15	0.49					
		VIF	1.69		1.82	1.27					
	Control	*r*	0.75		^a^	− 0.59	0.8	0.65	0.56	7.30	0.016
		*r*^2^	0.56			0.35					
		VIF	1.11			1.11					
Flexion velocity	COPD	*r*		^b^		− 0.76	0.76	0.58	0.53	11.15	0.01
		*r*^2^				0.58					
		VIF				–					
	Control	*r*		^c^		− 0.62	0.62	0.39	0.32	5.64	0.042
		*r*^*2*^				0.38					
		VIF				–					

Numerical data indicate the statistical parameters of the best model obtained by the backward stepwise regression. The model preceding the best model is indicated in the table by lowercase letters and the statistical parameters are the following: RF: Rectus Femoris; BF: Biceps Femoris; K: stiffness; B: viscosity; R: coefficient of correlation; *R*^2^: coefficient of determination; *aR*^2^: adjusted *R*^2^; *r*: partial coefficient of correlation; *r*^2^: partial coefficient of determination; VIF: Variance Inflation Factor; F: critical value for statistical significance; p: probability value for statistical significance.

^a^R = 0.8; F = 4,301; p = 0.051; ^b^R = 0.81; F = 3.986 p = 0.071; ^c^R = 0.75; F = 3.114; p = 0.084.

The RF activity and the level of passive tension were good predictors for the changes in velocity during leg extensions in both groups. However, considering the variables remained in the final models and their partial coefficients of correlation (*r*) and coefficient of determination (*r*^2^), the contribution derived from the RF activity was larger in the control group than in the COPD group, while stiffness and viscosity explained larger significant variances in COPD group than in the control group. Viscosity explained most of the variance during the flexion movements in both groups, with patients showing a contribution to the best model slightly larger than the control group (see rows related to *r*^2^ in Table 2). This regression analysis reveals that, with respect to controls, patients with COPD accomplished the natural active action integrating a smaller level of muscle activity with a higher contribution from passive components.

## Discussion

Patients with COPD, compared with healthy controls, exhibited reduced knee stiffness and viscosity when evaluated by passive pendular leg oscillations. Low passive viscoelastic tensions interacted with muscle activity during voluntary leg oscillations, providing an additional contribution in the response of the COPD group with respect to the control group.

In this study, the pendulum test was found to be a valuable tool in estimation the level of passive stiffness and viscosity of the knee musculoarticular complex, since no changes in muscle tone and no systematic phasic activations occurred. A similar lack in muscular activation was observed in the case of patients with rheumatoid arthritis^28^, but in that study an increase in stiffness and a reduction in kinematic parameters were detected due to the severe degeneration of joint tissues. Typically, in healthy individuals, isolated EMG activity can occur, particularly during the first leg flexion where a consistent muscle stretching can trigger reflex reactions. Systematic muscle reactions were also observed in individuals with low levels of stiffness and viscosity, such as in persons with Down Syndrome^25,29,30^. In the case of patients with COPD, the lack of muscle activity during the pendulum test may depend on a reduced muscle activity, as suggested by the lower level of EMG amplitude observed during the execution of active movements in patients with respect to healthy participants.

Impairments in skeletal muscle associated with COPD has been well documented^1,31^, while studies on passive tissue components have been restricted to pulmonary tissue^32^ or respiratory muscles^33^. No studies are known to us about the effects of COPD on the peripheral musculoarticular passive components.

Peripheral muscle dysfunctions in COPD are linked to the abnormal mechanical ventilation typical of this disease. In particular, a decrease of inspiratory capacity and an increase of end-expiratory lung volume, due to a dynamic hyperinflation of the lung, lead to a reduction in oxygen uptake, severe limitations of physical exercise and a progressive muscle disuse^1,31,34–36^. Tissue hypoxia and muscle disuse produce the typical peripheral muscle dysfunctions associated with COPD, consisting in muscle weakness and atrophy, enzymatic and mitochondrial abnormalities, and a shift from oxidative to fatigable muscular fibers^1,37^. However, long periods of skeletal muscle disuse may influence not only the active factors acting on joint movement, but also the passive viscoelastic components. This possibility was largely overlooked in the scientific literature addressing the musculoskeletal impairments associated with COPD. We believe that the physiological role of passive tension in joint biomechanics allows us to suppose that the reduction in viscoelastic tension, reported in this paper, may derive from the limited amount of physical exercise practiced by patients with COPD.

Muscle disuse may reduce viscoelasticity of the muscle-joint complex as reported by several studies. Loss of passive stiffness has been observed in human tendons after long periods (90–180 days) of simulated microgravity^38,39^, but also after 20 days of bed rest^40^. Muscle disuse also produces a decrease of intrinsic muscle passive tension by reducing the amount of titin, a noncontractile protein that is important for the structural stability of the sarcomere^41–43^.

Interestingly, DeBoer et al.^44^ demonstrated that both myofibrillar contractile proteins and tendon collagen synthesis rates decrease after 21 days of disuse, producing a reduction in cross-sectional area of quadriceps muscle and in patellar tendon stiffness. Reduced viscoelastic properties were also reported for the whole joint system, including not only passive muscle components, but also connective tissues, such as ligaments and capsules^45,46^.

Although muscle disuse is the most straightforward cause that can lead to a decrease of passive tension, other factors more directly connected with respiratory alterations may explain the results of the current study. A reduction in passive elastic components has been observed in lung tissue^32^ and in the ventilatory muscles^33^ of patients with COPD. Interestingly, in patients with mild to moderate COPD, changes in diaphragm titin isoform led to a reduction in passive tension generated by single fibers^33^. A possible association between changes in passive components observed for respiratory tissues and the reduction in viscoelastic properties, reported in the current study for the peripheral musculoarticular system, is a suggestive issue. Unfortunately, no experimental data are available to support this possibility, but it can be important to develop this topic in future studies.

A decrease in viscoelastic tension has relevant effects on joint biomechanics, reducing the level of mechanical resistance produced by passive tissues during joint rotation. In some conditions, low levels of stiffness and viscosity tension may be an advantage for joint mobility. It is the case of the active protocol used in this work where voluntary free leg oscillations were executed in the seated position. In fact, the low level of passive resistances compensated for the reduced muscle activity and permitted patients with COPD to move the leg with a velocity similar to that observed in healthy participants. In other cases, however, low passive resistances may reduce joint stability, affecting the mechanical work in most daily life motor activities, such as locomotion and upright standing.

Many studies showed that patients with COPD present difficulties in maintaining upright standing^13,47–51^ and stable walking^11,12,47,52,53^, with increased risk of falling^11,12,54–58^. Although the identification of specific causes for these motor disabilities is an open discussion^1^, most of the authors consider muscle weakness and limited oxygen supply as the main factors responsible for gait and balance alterations^13,47,50,54,59^. In this paper we report that the reduction in passive components in patients with COPD influenced both passive and active flexion–extension movements, as supported by the multiple regression analysis. Although no direct gait or balance test was performed by the participants of the current study, we believe that these findings may be generalized to natural everyday movements, taking into account that the effects of low viscoelastic tension depend on the type of motor task. In the case of balance skills, a reduction in passive resistance may decrease joint stability, leading to a more unstable level of balance in standing and walking. For example, a low elastic recoil during the push-off phase in the gait^14^ or a limited passive resistance when reacting to recover balance after posture perturbation^60–62^, reduce the contribution of mechanical work provided by passive components, deteriorating the ability to walk or stand. Thus, the harmful effect of COPD on these motor tasks may depend not only on muscle dysfunctions, but also a reduction in passive viscoelastic tension may play a role. Moreover, the deterioration of passive musculoarticular components may be of interest in understanding the mechanism determining the increase of risk of falling in patients with COPD. In fact, low viscoelastic tension worsens the balance ability more specifically in those contexts when the risk of falling is high, i.e. during transition tasks such as passing from sitting to standing, starting to walk or when standing while arm raising^12,47,49,62–64^.

Overall, considering the functional importance of passive components in movement biomechanics and the reduced viscoelastic tensions observed in this study in patients with COPD, it can be assumed that a decrease in passive musculoarticular tension may be an additional factor in determining postural and gait disorders associated with COPD. Since the current study was designed only to provide an accurate quantification to reveal significant changes in viscoelastic components in COPD, the hypothetical assumptions that viscoelastic reduction, reported in the current paper, may influence gait and balance dysfunctions should be verified by future experimental studies designed specifically for this purpose.

For a more comprehensive management of patients with COPD, this paper supports the idea that conventional pulmonary rehabilitation must be integrated with physical rehabilitation programs based on both muscle strength improvements and training aimed to increase viscoelastic tension of passive musculoarticular components. A variety of types and methodologies of training aimed at patients with COPD is reported in the scientific literature. Recent studies have shown that eccentric exercises, both during cycling^65^ and downhill walking^66^, improve muscle strength in COPD patients, enhancing whole-body exercise capacity to a similar extent of isotonic/isometric exercises. However, given the mechanical interaction between passive and active components observed in the current work, the joint torque produced during eccentric exercise is generated not only by the contractile components, but also by the engagement of the stretched parallel and serial elastic components of the muscle-joint complex^14,43,67^. Thus, when a reduction in passive viscoelastic tension occurs, a consequential therapeutic intervention might be to develop a rehabilitation program based on eccentric exercises. Our study suggests that the instrumented pendulum test can be a tool to evaluate objectively the level of stiffness and viscosity in patients with COPD, providing a help to monitor and personalize the rehabilitation interventions.

In conclusion, we believe that the changes in musculoarticular viscoelasticity observed in the current work provide novel insights into comprehension and interpretation of motor dysfunctions associated with COPD. However, additional studies are needed to better quantify the weight of the influence of passive musculoarticular components in producing specific motor task alterations.

This work has strengths, but also limitations. We quantified, for the first time, functional alterations the passive joint component. The new insights provided in this paper may stimulate a broader view of the effects of COPD on the musculoskeletal system. Furthermore, the pendulum test can be an easy tool to estimate the level of joint viscoelastic tensions.

However, two main limitations of the current study need to be considered.

First, a limited sample size should be considered in evaluating our results, especially for the multivariate analysis. In fact, as preliminary power analysis indicates that the sample size was adequate for the ANOVA and t-test, the sample size estimated for the multivariate analysis was slightly larger than the actual sample. One effect of a small sample size on multivariate regression models is that the difference between *R*^2^ and *aR*^2^ may increase, reducing the explanatory power of the model. The data in Table 3 indicate that this is not the case; in fact, the lag between the two measures was limited and the values of *aR*^2^ explain a good percent of the total variance accounted for by the models. Another measure that could be affected by a small sample size is the level of multicollinearity. High levels of multicollinearity may occur as the sample size reduces, producing inaccurate regression coefficients. The values of VIF found in the current study indicate that the multicollinearity in our models is within the range of acceptability and, thus, no substantial effect of sample size was detected on this measure^68^. Overall, we think that these tests can give the reader the opportunity to evaluate the goodness of the data reported in this study, confident that these finding can be reproduced using a larger sample.

Second, the experimental design did not include tests related to muscle functions (muscle strength, size and thickness) and to motor task functionality (gait and balance evaluations) that could have provided a greater clinical value to our data. At this stage, we chose to limit tests to those essential to reveal differences in passive components between COPD patients and healthy individuals. However, we implemented a simple motor functional test (active flexion–extension leg movements) that estimated the impact of the changes in passive components on a natural motor task.
